# Causality, Severity, Preventability and Predictability Assessments Scales for Adverse Drug Reactions: A Review

**DOI:** 10.7759/cureus.59975

**Published:** 2024-05-09

**Authors:** Pramod K Manjhi, Madhusudan P Singh, Mukesh Kumar

**Affiliations:** 1 Pharmacology, All India Institute of Medical Sciences, Patna, Patna, IND; 2 Pharmacology, All India Institute of Medical Sciences, Raipur, Raipur, IND; 3 Pharmacology, Narayan Medical College and Hospital, Sasaram, IND

**Keywords:** pharmacovigilance, adverse drug reaction reporting, preventability assessment, severity assessment, causality assessment

## Abstract

The pharmacovigilance program of India (PvPI), after its inception, has been reliably acquiring force in bringing issues to light among the masses, healthcare professionals, the pharma industry, and clinical staff at hospitals. Adverse drug reactions are unintended events that occur after exposure to a drug, biological product, or medical device, and they may result in morbidity and mortality. It is critical to monitor the safety of drugs during the post-marketing phase to find long-term and rare ADRs, as well as ADRs in special populations and patients with co-morbidities that are not usually included during clinical trials. The definitive objective of pharmacovigilance is to collate data and analyze it. Assessing the causality between ADRs and drugs is necessary to decrease the occurrence of ADRs and to reduce the risk of drug-related ADRs. ADRs may lead to increased morbidity, increased hospital stays, and increased cost of treatment, resulting in compromised patient safety. Causality assessment is the evaluation of the likelihood that a particular treatment is the cause of an observed adverse event and establishing a causal association between a drug and a drug reaction is necessary to prevent further recurrences. Numerous methods available for establishing a causal association between the drug and adverse events have been broadly classified into clinical judgment or global introspection, algorithms, and probabilistic methods. These include the Swedish method, World Health Organization-Uppsala Monitoring Centre (WHO-UMC) scale, Naranjo's algorithm, Kramer algorithm, Jones algorithm, Karch algorithm, Bégaud algorithm, Adverse Drug Reactions Advisory Committee guidelines, Bayesian Adverse Reaction Diagnostic Instrument, and so on. Despite various methods available, none of the causality assessment tools have been universally accepted as the gold standard. Naranjo's algorithm and WHO-UMC scales are, however, the most commonly used. Similarly, for preventability and severity assessment of ADRs, the Schumock and Thornton scale and Hartwig and Siegel's scale are most commonly used. Hence, we reviewed different tools and methods available to assess the causality, preventability, and severity of ADRs.

## Introduction and background

Pharmacovigilance refers to the science and activities focused on identifying, evaluating, comprehending, and preventing adverse drug reactions (ADRs) and other drug-related problems [1]. Its purpose is to establish a link between drug exposure and the occurrence of ADRs and to assess drug safety across populations. The World Health Organization defines an ADR as "an unintended and noxious reaction to a drug that occurs at doses typically used for disease diagnosis, prophylaxis, or therapy, or to modify physiological function" [2]. In other words, an ADR is a harmful effect that directly results from using a drug at standard therapeutic doses.

ADRs are often underreported globally, and there are several reasons for this underreporting [3]. Physicians are primarily focused on treating patients rather than reporting ADRs, and the nature of intensive care unit work can make it challenging to identify ADRs [4]. Additionally, it can be difficult to distinguish ADRs from symptoms or consequences of underlying medical conditions. Furthermore, treating physicians may not always recognize the correlation between changes in biochemical parameters, haematological profile, and ECG changes with drug-related ADRs. For example, in a case report published by Buch and Anderson in 2015, a patient developed QT prolongation after being prescribed a combination of fluconazole, clarithromycin, and amiodarone, which are known to prolong the QT interval [5]. This ADR went unrecognized initially, highlighting the importance of pharmacovigilance in identifying such events.

The active participation of doctors and other healthcare staff is critical in ADR reporting and pharmacovigilance. Undetected ADRs can result in prolonged hospitalization, increased medical expenses, worsened health outcomes for patients, and higher costs for the healthcare system. For instance, a study published in the New England Journal of Medicine found that ADRs were a leading cause of hospitalization and accounted for a substantial proportion of in-hospital costs [6]. An ADR monitoring system that follows a logical and scientific approach can greatly enhance the health and well-being of the population. Moreover, Pharmacovigilance has the potential to identify substandard quality drugs and prevent drug prescribing, dispensing, and administration errors, making it an essential long-term goal [1]. 

Conducting a causality assessment to establish or evaluate the link between ADRs/Adverse events (AEs) and medications is crucial for reducing the incidence of such events and minimizing patients' exposure to potential medication risks [7]. A notable example is the case of rofecoxib (Vioxx®), a non-steroidal anti-inflammatory drug that was withdrawn from the market in 2004 after pharmacovigilance efforts revealed an increased risk of cardiovascular events associated with its use [8]. This assessment is a vital aspect of pharmacovigilance and contributes to the evaluation of risk-benefit ratios and the identification of signals, thereby assisting in regulatory decision-making processes [9].

Causality assessment is a useful tool for evaluating and analysing case reports, relying on a comprehensive assessment of the clinical-pharmacological aspects of the patient's history and the quality of observation documentation [10]. To achieve its objectives, causality assessment relies on four fundamental criteria, including temporal eligibility, de-challenge and outcome, re-challenge and outcome, and confounding factors [11]. In this review, we explore the different causality scales that are available and their applicability in real-world clinical practice.

## Review

Causality assessment provides several significant benefits, such as reducing evaluators' disagreements, determining the probability of a relationship, facilitating the assessment of individual case reports, and improving scientific evaluation and education [12]. However, there are certain limitations to this method, such as the inability to differentiate between valid and invalid cases and the difficulty in quantitatively measuring the likelihood of a relationship.

Methods of causality assessment and their necessity

Causality assessment is a crucial aspect of drug safety evaluation, as it involves identifying adverse drug reactions (ADRs) and determining their causal relationship with the administered drug. This process is essential for the development and use of safe medications. Various researchers and institutions have developed several methods and tools for causality assessment based on different criteria; however, no single method has gained universal acceptance [12].

The criteria for causality assessment include a chronological relationship between drug administration and the occurrence of an adverse event, confirmation of the event and its evidence through in vitro or in vivo tests, screening for other drug or disease-related causes, and considering previous history of similar events [7]. These methods can be broadly classified into three categories: expert judgment or global introspection tools, algorithmic tools, and probabilistic tools [13]. Expert judgement or global introspection tools involve the evaluation of an adverse event by an expert or group of experts, considering all the relevant available data. This evaluation is based on retrospective data and expert knowledge on the subject. Expert judgement is often used when there is insufficient data to apply an algorithmic or probabilistic approach [14]. For instance, in a case study published in the Journal of Pharmacovigilance, a panel of experts determined that a rare skin reaction observed in a patient was likely caused by a newly approved medication despite limited data on the drug's adverse event profile [15].

Algorithmic tools employ a systematic approach involving a structured and standardized questionnaire form with a flowchart and stepwise instructions to conclude the causality of specific adverse events. The questionnaire primarily derives data regarding the temporal sequence, time of onset of the adverse event, previous history of events, de-challenge (improvement after drug withdrawal), and rechallenge (recurrence of the event upon reintroduction of the drug) [14]. One widely used algorithmic tool is the Naranjo Adverse Drug Reaction Probability Scale, which assigns scores based on these criteria to determine the likelihood of an ADR [13].

Probabilistic or Bayesian tools are based on prior and posterior probabilities. Prior probability is calculated from epidemiological data, while posterior probability is calculated from both the evidence and epidemiological data [16]. This method facilitates the simultaneous assessment of multiple potential causes of adverse events. For example, the Bayesian Adverse Reaction Diagnostic Instrument (BARDI) has been used to evaluate the causality of adverse events in clinical trials by integrating prior knowledge and observed data [17]. It is crucial to note that the choice of method or tool for causality assessment depends on the specific situation and available data. No single method or tool can be universally applied to all situations, and the expertise of a trained evaluator is essential to make an accurate assessment of the causality of an adverse event [18]. Table 1 lists various tools or scales that come under the above categories.

**Table 1:** Example of different tools/scales available for causality assessment

Expert judgement or global introspection tools	Algorithmic tools	Probabilistic tools
WHO-UMC Causality Assessment Criteria	Naranjo Scale	Bayesian adverse reaction diagnostic instrument (BARDI)
Swedish method by Wilholm et al.	Roussel Uclaf causality assessment method (RUCAM)	Australian method
	Dangaumou’s French method	MacBARDI spreadsheet
	Kramer et al. method	
	Balanced assessment method	
	Drug interaction probability scale (DIPS)	
	Summary time plot	
	Ciba Geigy method	
	Maria and Victorino (M & V) scale	
	Karch and Lasagna tool	
	Priscus List	
	Liverpool ADR Scale	
	Hallas Scale	

Expert judgement or global introspection tools XPERT

The WHO-UMC Causality Assessment System

The World Health Organization-Uppsala Monitoring Centre (WHO-UMC) framework is a widely accepted and globally recognized system for the evaluation of case reports related to adverse drug reactions (ADRs) caused by suspected medications [19]. This framework has been developed in consultation with the National Centres participating in the Programme for International Drug Monitoring and is considered a practical and pragmatic tool for the causality assessment of ADRs [20].

The WHO-UMC causality framework is a consolidated evaluation that considers the clinical-pharmacological aspects of the case history and the quality of documentation of perception [7]. The WHO scale is a commonly used scale for assessing the causal relationship between adverse drug reactions and suspected products, leading to scientific evaluation [13]. The WHO causality scale provides a structured and standardized assessment of causality, and it is divided into six levels based on four criteria. These criteria include the plausible time-event relationship between drug use and adverse events, the absence of other competing causes such as medications or diseases, the response to drug withdrawal or dose reduction (de-challenge), and the response to drug re-administration (re-challenge) [7]. Based on the fulfilment of these criteria, causality assessment is done into a different level of causality association.

If there is insufficient or contradictory information in the case report, it can be labelled as un-assessable/unclassifiable. In contrast, if additional data are needed for assessment, the report is classified as conditional/ unclassified [7]. The WHO-UMC framework is considered a practical and reliable tool for the evaluation of case reports, and the WHO causality scale is a commonly used tool for assessing the causal relationship between adverse drug reactions and suspected products [12]. Table 2 lists the various causality categories with their descriptions and an explanation.

**Table 2:** WHO-UMC causality assessment scale WHO-UMC: World Health Organization-Uppsala Monitoring Centre

Causality term	Causality term
Certain	· Event or laboratory test abnormality, with a plausible time relationship to drug intake
	· Cannot be explained by disease or other drugs
	· Response to withdrawal plausible (pharmacologically, pathologically)
	· Event definitive pharmacologically or phenomenologically (i.e., an objective and specific medical disorder or a recognised pharmacological phenomenon)
	· Rechallenge satisfactory, if necessary
Probable / Likely	· Event or laboratory test abnormality, with reasonable time relationship to drug intake
	· Unlikely to be attributed to disease or other drugs
	· Response to withdrawal clinically reasonable
	· Rechallenge not required
Possible	· Event or laboratory test abnormality, with reasonable time relationship to drug intake
	· Could also be explained by disease or other drugs
	· Information on drug withdrawal may be lacking or unclear
Unlikely	· Event or laboratory test abnormality, with a time to drug intake that makes a relationship improbable (but not impossible)
	· Disease or other drugs provide plausible explanations
Conditional / Unclassified	· Event or laboratory test abnormality
	· More data for proper assessment needed, or
	· Additional data under examination
Un-assessable / Unclassifiable	· Report suggesting an adverse reaction
	· Cannot be judged because the information is insufficient or contradictory
	· Data cannot be supplemented or verified

Practical Application and Case Examples

The practical application of the WHO-UMC Adverse Drug Reaction (ADR) scale framework spans various real-world scenarios, offering a structured approach to evaluating the likelihood of drug-related adverse events. For example, in a study conducted at the medicine outpatient department of the 150-bed Majeedia Hospital, Hamdard University Campus in New Delhi [21], researchers aimed to monitor adverse drug reactions linked to antihypertensive medications. Utilizing the WHO-UMC criteria, registered pharmacists conducted one-to-one patient interviews, employing a questionnaire-based Adverse Drug Reaction Monitoring Form aligned with World Health Organization guidelines. Over a four-month period, the study identified 34 adverse drug reactions among 250 hypertensive patients. Notably, a significant proportion of these reactions occurred among middle-aged and female patients, with cardiovascular-related adverse events being predominant. The analysis highlighted beta-blockers as the drug category most frequently associated with adverse reactions, followed by angiotensin-converting enzyme inhibitors and calcium channel blockers. This study underscores the practical utility of the WHO-UMC framework in comprehensively assessing adverse drug reactions, thereby aiding in optimizing patient care strategies [21].

Validation Studies and Reliability

Numerous validation studies have investigated the reliability and validity of the WHO-UMC framework. A systematic review conducted by Agbabiaka et al. [12] scrutinized various causality assessment methodologies, including the WHO-UMC scale, affirming its reliability and widespread acceptance in evaluating ADRs. Additionally, Gallagher et al.'s [22] study showcased strong inter-rater agreement and validity of the WHO-UMC scale [7] across a diverse array of case reports.

Challenges and Limitations

Despite its utility, the WHO-UMC framework is not without challenges and limitations. Inter-rater variability can arise, particularly in instances where information is incomplete or ambiguous [23]. Moreover, assessing causality for certain adverse events, such as delayed or chronic reactions, may prove intricate and necessitate careful consideration of additional factors [24].

Despite these challenges, the WHO-UMC framework retains its status as a widely recognized and practical tool for assessing ADR causality associated with suspected medications. Ongoing research endeavours and refinements can further augment its applicability while addressing potential limitations.

Swedish method by Wilholm BE

The Swedish method, developed by Wilholm BE [25], is a causality assessment tool that considers seven factors to evaluate the relationship between an adverse drug reaction and the use of a drug. These factors include the time sequence of cause and effect, past information on the drug, dose-response relationship, response pattern to the drug, re-challenge, alternative cause of aetiology, and concomitant drugs.

Based on these factors, events are classified into probable or possible categories, or as non-assessable or unlikely. The Swedish method has a limited number of classes for causality classification, which can lead to incorrect categorization and assessment. Despite its limitations, this method is widely used and has been implemented in several countries, including Sweden and Norway [25]. One of the strengths of the Swedish method is its simplicity and ease of use. It requires minimal information and can be applied to a wide range of cases. However, its reliance on a limited set of factors can also be a weakness, as it may not take into account all relevant information and may lead to incorrect conclusions [25].

Algorithmic tools

Naranjo’s Scale

This method is widely accepted and is used to determine the likelihood of whether the adverse event is due to the drug or other factors. The Naranjo's Scale comprises of 10 questions, each answered as “Yes”, “No”, or "Do not know," with distinctive point values assigned to each response. However, the method does not consider drug-drug interactions, and it only assesses one drug at a time for causality. Furthermore, the score is subtracted if another factor is responsible for an adverse event, thereby weakening the causal association. The validity and reliability of this tool have not been confirmed in children [13,26,27]. The questionnaire is a structured and standardized approach, and a simplified version of the questionnaire can be found in Tables 3-4.

**Table 3:** Naranjo’s adverse drug reaction probability scale questionnaires

Sr. No.	Please answer the following questionnaire and give the pertinent score	Yes	No	Do Not Know	Score
1.	Are there previous conclusive reports on this reaction?	1	0	0	
2.	Did the adverse drug reaction occur after the administration of the suspected drug/medication?	2	-1	0	
3.	Did the adverse drug reaction improve when the suspected drug was discontinued or a specific antagonist was administered?	1	0	0	
4.	Did the adverse drug reaction reappear after Re-administration of the suspected drug/medication?	2	-1	0	
5.	Are there other possible alternative causes that could have, on their own, caused the reaction?	-1	2	0	
6.	Did the reaction reappear on the administration of a placebo?	-1	1	0	
7.	Was the drug detected in the blood (or other fluids) in toxic concentrations?	1	0	0	
8.	Was the reaction more severe when the dose was expanded or then again less severe when the dose was diminished?	1	0	0	
9.	Did the patient have a similar reaction to a similar drug or a related agent in any past drug history?	1	0	0	
10.	Was the adverse drug reaction confirmed by any objective evidence?	1	0	0	

**Table 4:** Naranjo’s adverse drug reaction probability scale scoring

Causality and Score	Explanation of Score
Definite: If the total score is 9 or greater than 9	The adverse reaction concerned with suspected drug followed reasonable objectives in which either drug detected in the blood (or other fluids) have toxic Concentrations. Adverse drug reaction occurs after the administration of suspected drug/medication and reaction reappear on the administration of placebo or drug reaction improve when the suspected drug was withdrawn.
Probable: If the score is in between 5-8	The adverse reaction concerned with the suspected drug followed reasonable objectives in which adverse drug reaction occurs after the administration of the suspected drug/medication with recognised response confirmed by drug withdrawal. Not depend upon the clinical status of the patient.
Possible: If the score is in between 1-4	The adverse reaction followed a temporal sequence after drug exposure, possibly followed a recognized pattern to the suspected drug., and could be explained by characteristics of the patient's disease.
Doubtful: If the score is 0	The adverse reactions has their alternative causes other than the suspected product.

Roussel Uclaf Causality Assessment Method (RUCAM)

The Roussel Uclaf Causality Assessment Method (RUCAM) is a widely used tool to determine the link between a drug and an adverse drug reaction (ADR) [28]. It is not limited to assessing drug-induced liver injury (DILI), but can also be used to identify other drug-related adverse events such as cutaneous reactions and drug-induced acute pancreatitis [28].

RUCAM assesses the strength and quality of evidence that connects the drug to the ADR. It provides a scoring system ranging from -5 to +14, which classifies the causality of the ADR as unlikely, possible, probable, or highly probable. A higher score indicates a greater likelihood that the drug caused the adverse event [28-30]. One of the advantages of RUCAM is that it takes into account multiple factors to evaluate causality, including the timing of the adverse event relative to drug use, the response to re-exposure, the absence of alternative causes, and previous knowledge of the drug's toxicity. However, one of the limitations of this tool is that it requires expert judgment and is not entirely objective [28] (Table 5).

**Table 5:** Roussel Uclaf causality assessment scale

Criteria	Description	Score	Patient’s Score
Time to onset of infection	5-90 days of drug start	+2	
	<5 or > 90 days of drug start	+1	
	≤15 days from drug cessation	+1	
Course of reaction after drug cessation	Decrease ≥50% within 8 days	+3	
	Decrease ≥50% within 30 days	+2	
	Not application	+1	
	No information/Decrease ≥50% after 30 days	0	
	Recurrent increase	-2	
Risk factors	Age ≥55	+1	
	Alcohol use	+1	
Concomitant drugs	Time to onset incompatible	0	
	Time to onset compatible but unknown reaction	-1	
	Time to onset compatible but known reaction	-2	
	Role proved in this case	-3	
	None or information not available	0	
Non drug related causes	All causes reasonably ruled out	+2	
	6 causes of group 1 ruled out	+1	
	5 or 4 causes of group 1 ruled out	0	
	Less than 4 causes of group 1 ruled out	-2	
	Non drug cause highly probable	-3	
Previous information on drug	Reaction unknown	0	
	Reaction published but unlabelled	+1	
	Reaction labelled in product characteristics	+2	
Response to re-administration	Positive	+3	
	Compatible	+1	
	Negative	-2	
	Not available or Not interpretable	0	

Dangaumou's French Method

Dangaumou's French method is a clinical tool widely used in assessing the causality of adverse drug reactions (ADRs) in patients [31]. Developed in France, it relies on the evaluation of several clinical criteria to determine the probability of a drug causing an ADR. These criteria include the onset of the ADR, the patient's clinical evolution after drug administration, laboratory test results, and other factors that could explain the ADR [31]. Additionally, the method considers the patient's previous history and the drug's known adverse effects. One of the primary benefits of Dangaumou's French method is its ease of use and the lack of specialized knowledge required. However, some of its limitations include subjective interpretation of the criteria and difficulty in distinguishing between the drug's effects and those of the underlying disease [31,32]. 

Dangaumou's French method is a tool used to assess suspected drugs along with other drugs taken at the same time. It takes more time to use than other causality assessment scales. The scores are grouped into likely, possible, and dubious. This method uses seven criteria in two different tables. Three chronological and four semiological criteria are used. Chronological criteria include drug challenge, de-challenge, and rechallenge. Semiological criteria include suggestive clinical signs, favouring factors, alternate non-drug-related explanations, and specific laboratory tests with three possible outcomes. This method helps distinguish intrinsically abused substances from bibliographical data. Nevertheless, Dangaumou's French method remains a valuable tool for clinicians in the assessment of ADRs in patients [31,33,34].

Kramer Method

The Kramer Method is a widely used tool for healthcare professionals to assess the likelihood of a drug causing an adverse drug reaction (ADR) [35]. Developed by Dr. Mark Kramer, the method involves a comprehensive analysis of all available evidence, including the patient's medical history and laboratory tests. It aims to determine the strength of the relationship between the drug and the ADR and classifies causality as certain, probable, possible, or unlikely [36]. The Kramer Method is flexible and adaptable, making it suitable for various ADRs and clinical situations. However, it requires clinical expertise and access to detailed patient data, and it may be subject to interpretation bias. Despite its limitations, the Kramer Method is valuable in improving patient safety, reducing the risk of ADRs, and improving patient outcomes [35].

In a study by Mertens et al., the adjusted Kramer algorithm was evaluated for its ability to assess causality of drug-related admissions (DRAs) in geriatric inpatients, a significant cause of preventable harm in older adults. Compared to the Naranjo algorithm, the adjusted Kramer algorithm demonstrated a higher positive agreement with expert consensus in determining DRA causality. Diuretics were identified as the main culprits of DRAs, with falls being the most commonly attributed adverse event. The study suggests the implementation of the adjusted Kramer algorithm as part of a standardized assessment for DRAs in older adults [35,36]. 

Balanced Assessment Method

The Balanced Assessment Method (BAM) is a causality assessment tool used to evaluate the likelihood of a drug causing an adverse drug reaction (ADR) [37]. The method was developed by the European Medicines Agency (EMA) and uses a systematic approach to evaluate evidence. The BAM consists of five steps, including defining the problem, collecting information, analysing data, interpreting results, and communicating findings. The method considers various factors such as the drug's nature, pharmacokinetics and pharmacodynamics, the clinical context of the ADR, and other risk factors. BAM assigns the highest weight to the temporal relationship between the drug and the ADR and the lowest to anecdotal reports [37]. One of the strengths of BAM is its objective and transparent approach to causality assessment, making it useful for pharmacovigilance activities. However, it may not be suitable for complex or unknown mechanism ADRs and requires detailed clinical and laboratory data, which may not be available in all settings [37].

Drug Interaction Probability Scale (DIPS)

The Drug Interaction Probability Scale (DIPS) is a widely-used tool to evaluate the probability of drug interactions occurring [38]. The scale ranges from 0 to 13, with higher scores indicating a higher likelihood of interaction. The DIPS was originally developed by Horn and Hansten in 1979 as a method to evaluate drug-drug interactions. 

To assess the likelihood of drug interactions, the DIPS considers four criteria, including the inherent pharmacological activity of the drugs, the patient's clinical status, the time course of the interaction, and the similarity of the interaction to previously reported cases [38]. Each criterion is evaluated on a scale of 0 to 3, with a score of 0 indicating no interaction and a score of 3 indicating a highly probable interaction. The total score is calculated by adding up the scores for each criterion, with a maximum score of 13 indicating a highly probable interaction [38]. The DIPS has broad applications, and it can be used in clinical practice, research studies, and regulatory reviews to evaluate drug interactions. Additionally, it can assist healthcare professionals in identifying potential drug interactions before prescribing a medication. Although the DIPS is a useful tool, it does have some limitations. It depends on the availability of information about the drugs in question and the patient's clinical status, which may not always be readily available. Moreover, it does not consider individual patient factors that may influence the probability of a drug interaction [38] (Table 6).

**Table 6:** Drug interaction probability scale (DIPS)

Question	Answer	Score
Are there previous credible reports of this interaction in humans?	Yes	+1
Is the observed interaction consistent with the known interactive properties of the precipitant drug?	Yes	+1
Is the observed interaction consistent with the known interactive properties of the object drug?	Yes	+1
Does the interaction remit on the dechallenge of the precipitant drug with no change in the object drug?	Yes	+1
Did the interaction reappear when the precipitant drug was readministered in the presence of continued use of the object drug?	Unknown	0
Are there reasonable alternative causes for the event?	No	+1
Was the object drug detected in blood or other fluids in concentrations consistent with the proposed interaction?	Yes	+1
Was the drug interaction confirmed by any objective evidence consistent with the effects on the object drug?	No	0
Was the interaction greater when the precipitant drug dose was increased or less when the precipitant drug dose was decreased?	Unknown	0
Total DIPS score		6

In a study by Bindler et al., the Drug Interaction Probability Scale (DIPS) was applied as an objective tool to assess the likelihood of drug-drug interactions (DDIs) in a two-day pilot protocol evaluating the addition of smoked cannabis to orally administered hydrocodone/acetaminophen combination products in a patient with chronic pain diagnoses. The study demonstrated the utility of the DIPS in objectively evaluating the causation of DDIs, with a probable interaction observed between orally administered hydrocodone/acetaminophen and inhalation of combusted cannabis based on the standard set of 10 questions. Despite the complexity of clinical scenarios involving multiple medical conditions and prescribed medications, the DIPS facilitated the identification of potential DDIs, underscoring its importance in aiding healthcare professionals in making informed decisions regarding patient care and medication management [39].

Summary Time Plot

The summary time plot is a widely used graphical tool in the pharmaceutical industry that enables analysis of the relationship between drugs and adverse drug reactions (ADRs) based on time factors [40]. By plotting the onset lag time between drug administration and the occurrence of an adverse event on the X-axis, and the severity of the adverse event on the Y-axis, the summary time plot summarizes the plausible relationship between drugs and ADRs [40].

One of the benefits of using a summary time plot is that it saves time and resources while providing reliable results using legally acceptable terminology. Additionally, it can identify potential drug safety issues early on in the drug development process. However, it is important to note that the summary time plot only considers the time factor and cannot provide a conclusive analysis based on other factors [40]. Moreover, the summary time plot is a valuable tool that can help identify patterns and occurrences of ADRs over time. This information can assist in determining if a drug is causing an adverse event or if the event is unrelated to the drug. It can also be used to identify potential risk factors for ADRs, such as age or gender. Despite its benefits, the summary time plot has some limitations. It requires a substantial amount of data to be effective and may not be useful in cases where there is limited data available. It also cannot provide a conclusive analysis of ADRs and drug safety based solely on the time factor [40].

Ciba Geigy Method

The Ciba Geigy method, developed by the pharmaceutical company Ciba-Geigy (now Novartis), is a widely-used pharmacovigilance technique that assesses the relationship between drugs and adverse drug reactions (ADRs) [41]. The method follows a three-step process to evaluate the association between drugs and ADRs. In the first step, a team of experts reviews all available data on the drug, including clinical trials, post-marketing surveillance data, and case reports. The team then identifies all ADRs associated with the drug and assigns each ADR a severity level. In the second step, the team evaluates the causal relationship between the drug and each ADR. This involves analyzing the temporal relationship between the drug and the ADR, as well as any other factors that may have contributed to the ADR. The team assigns a causal relationship score to each ADR based on their findings. In the final step, the team assesses the clinical significance of each ADR. This involves considering the severity and frequency of the ADR, as well as its potential impact on patient outcomes. The team then assigns a clinical significance score to each ADR [41].

One of the main strengths of the Ciba Geigy method is that it takes into account multiple factors when evaluating the relationship between drugs and ADRs. It also follows a standardized approach that promotes consistency in evaluating drugs across different therapeutic areas. However, the method has some limitations. The process can be time-consuming and resource-intensive, as it requires a team of experts to review all available data on a drug. Additionally, subjective assessments can introduce variability in the evaluation process, potentially leading to inconsistencies in the findings [41].

Maria and Victorino Scale for Causality Assessment

The Maria and Victorino Scale, created by Dr. Maria and Dr. Victorino in 1998 and published in the European Journal of Clinical Pharmacology, is a popular method used to determine the causality of adverse drug reactions (ADRs) [42,43]. It assesses the probability of a causal relationship between a drug and an adverse event by assigning a score ranging from -5 to +5. The criteria used for the rating include the time between drug administration and the onset of the adverse event, the de-challenge and re-challenge effects, and other factors such as co-morbidities, concomitant medications, and patient characteristics. Assigning a score to each criterion helps healthcare professionals to make informed clinical decisions.

However, it is important to note that the Maria and Victorino Scale is not a definitive measure of causality and should be used in conjunction with clinical judgment and other available evidence [43]. The scale also does not consider the severity of adverse events or the potential benefits of drug therapy. Therefore, it should be used as part of a comprehensive approach to pharmacovigilance that includes ongoing monitoring and reporting of adverse events. Despite these limitations, the Maria and Victorino Scale remains a valuable tool for assessing adverse drug reactions and promoting patient safety due to its broad applicability and ease of use [43] (Table 7).

**Table 7:** Maria and Victorino scale for causality assessment

Maria and Victorino criteria	Score
Chronology Criterion	
From drug intake until event onset	+1 to +3
From drug withdrawal until event onset	-3 to +3
Time-course of the reaction	0 to +3
Exclusion of alternative causes	-3 to +3
Extrahepatic manifestations	0 to +3
Literature data	-3 to +2
Re-challenge	0 to +3
Scores: >17 points: Definite 14-17: Probable 10-13: Possible 6-9: Unlikely <9: Excluded	

Karch and Lasagna Scale

The Karch and Lasagna scale is a method used to evaluate the causality of adverse drug reactions (ADRs). It was first introduced by Karch and Lasagna in 1977 and is still widely used today. This scale involves examining the temporal relationship between the administration of the drug and the onset of the adverse event, as well as other factors that may be contributing to the reaction [44].

The Karch and Lasagna scale considers four main criteria: temporal relationship, de-challenge, re-challenge, and alternate causes [44]. Temporal relationship refers to the time between drug administration and the onset of the adverse event. If the reaction occurs shortly after administration, it is more likely to be related to the drug. De-challenge refers to the resolution of the adverse event after discontinuation of the drug. If the adverse event disappears or improves after the drug is stopped, it is more likely to be related to the drug. Re-challenge involves re-administration of the drug after the adverse event has resolved, and the recurrence of the adverse event. If the reaction recurs after re-administration, it is more likely to be related to the drug. Lastly, alternate causes are considered, such as other medications, underlying medical conditions, or environmental factors that could contribute to the adverse event [44].

The Karch and Lasagna scale assigns scores ranging from 0 to 9, with higher scores indicating a greater likelihood of a causal relationship between the drug and the adverse event. Scores of 0-1 indicate that the reaction is unlikely to be related to the drug, while scores of 7-9 suggest a high probability of causality [44]. This scale has been used in clinical trials and pharmacovigilance to assess the safety of drugs and to identify potential adverse reactions. This scale has some limitations. The scale does not consider the severity of the adverse event or the potential benefits of the drug therapy. It also relies on the subjective judgment of the healthcare professional using it, which can lead to variability in interpretation. Despite these limitations, the Karch and Lasagna scale remains a valuable tool for evaluating ADRs, particularly in situations where there is limited information available (Table 8).

**Table 8:** Karch and Lasagna algorithm

Karch and Lasagna algorithm	
Minor	No antidote or treatment is required; hospitalization is not prolonged.
Moderate	A change in the treatment (e.g., modified dosage, the addition of drug), but not necessarily discontinuation if the drug is required; hospitalization be prolonged, or specific treatment may be required.
Severe	ADR may be life-threatening and requires immediate discontinuation of the drug and specific treatment for ADR
Lethal	Contributes to patient's death

Priscus List

The PRISCUS List is a collection of drugs that may be unsuitable for use in elderly patients due to their potential to cause adverse drug events (ADEs) [45]. ADEs occur when a patient experiences harm or injury as a result of taking a medication. This is a significant concern in elderly patients, who are more susceptible to the harmful effects of drugs due to their aging bodies and altered pharmacokinetics and pharmacodynamics. The list was developed in Germany in 2010 through a systematic review of available evidence and expert consensus. The medications on the list have been classified into three categories based on their risk of causing ADEs in elderly patients [45]. The first category is drugs to avoid in all cases, including long-acting benzodiazepines, first-generation antihistamines, and tricyclic antidepressants. The second category is drugs to avoid in most cases, including proton pump inhibitors, non-steroidal anti-inflammatory drugs (NSAIDs), and selective serotonin reuptake inhibitors (SSRIs). Although these medications may be necessary in some circumstances, they should be avoided whenever possible due to their high risk of ADEs in elderly patients. The third category is drugs to use with caution, including angiotensin-converting enzyme (ACE) inhibitors, diuretics, and anticoagulants. These medications have a moderate to high risk of causing ADEs in elderly patients and should be closely monitored to reduce the risk of harm [45].

It is important to note that the PRISCUS List is not a definitive guide to prescribing medications for elderly patients. Rather, it is a tool to assist healthcare providers in making informed decisions about medication use in this population. Healthcare providers should consider individual patient characteristics and factors when making prescribing decisions and weigh the potential benefits and risks of medication use. In conclusion, the PRISCUS List is a valuable resource for healthcare providers in identifying potentially inappropriate medications for elderly patients. By avoiding or using these medications with caution, healthcare providers can reduce the risk of ADEs and improve patient outcomes [45].

Liverpool ADR Avoidability Assessment Tool

The Liverpool ADR Avoidability Assessment Tool is a tool used to determine whether an ADR could have been avoided or not. Developed by researchers at the University of Liverpool, the tool is based on the World Health Organisation's (WHO) classification of ADRs as Type A or Type B [21]. The Liverpool ADR Avoidability Assessment Tool consists of a series of questions designed to evaluate the likelihood that an ADR could have been avoided. The questions consider factors such as the appropriateness of the medication's prescription, whether the patient was adequately monitored, and whether the ADR was a predictable consequence of the medication's pharmacology. This tool is particularly helpful in identifying the root causes of ADRs and developing strategies to prevent them from happening in the future [21]. It can also help healthcare providers assess their prescribing practices and identify areas for improvement. However, the Liverpool ADR Avoidability Assessment Tool requires a significant amount of knowledge and expertise from the healthcare provider using it. An understanding of the medication's pharmacology and the patient's medical history is necessary to determine whether the ADR could have been avoided or not [21].

Hallas Scale 

The Hallas Scale of Adverse Drug Reactions (ADRs) is a tool used to quantify the severity of ADRs. It was developed by Morten Hallas in 1993 and is widely used in clinical research [46]. The Hallas Scale rates the severity of ADRs on a five-point scale, ranging from 0 to 4. The severity of the ADR is determined by the healthcare provider based on the patient's symptoms and clinical course. A score of 0 indicates no ADR, while a score of 4 indicates a severe ADR that requires hospitalization or causes permanent disability or death. Scores 1, 2, and 3 indicate mild, moderate, and severe ADRs that require no intervention, some intervention, or hospitalization, respectively [46].

The Hallas Scale has been validated in numerous studies and is a reliable tool for assessing the severity of ADRs. It is particularly useful in clinical research to determine the impact of medications on patient outcomes [46]. One limitation of the Hallas Scale is that it does not consider the preventability of ADRs. It only evaluates the severity of the ADR and does not take into account whether the ADR could have been avoided with different prescribing practices. In conclusion, the Hallas Scale of ADRs is a valuable tool for quantifying the severity of ADRs. Its use in clinical research can help assess the impact of medications on patient outcomes. However, it should be used in conjunction with other tools for evaluating the preventability of ADRs and their impact on patient health [46].

Probabilistic tools

Bayesian Methods

The Bayesian method is a statistical approach used to determine the cause of an adverse drug reaction. This method involves using the Bayesian Adverse Reaction Diagnostic Instrument (BARDI), which combines prior knowledge from earlier epidemiological studies with current case data provided by a clinical specialist [47]. By doing so, it calculates the posterior probability or posterior odds of the drug causing a specific adverse drug reaction. The probability ratio is based on factors such as the patient's medical history, the timing of the adverse event in relation to the drug, and any signs or symptoms that occurred after the drug was withdrawn or re-administered [47]. However, Bayesian techniques can be complex and involve cumbersome computations, requiring the use of Bayesian spreadsheets. To help with the assessment of cutaneous adverse drug reactions (CADRs), dermatologists can use causality evaluation tools like the WHO-UMC scale, which provides a more detailed and effective pharmacovigilance approach to dealing with adverse drug reactions [47].

MacBARDI Spreadsheet

The MacBARDI spreadsheet is a computerized version of the BARDI method, which is used for causality assessment [48,49]. The MacBARDI tool is divided into three main sections, which include a spreadsheet with database access, a knowledge base that is integrated with clinical features, and a set of limited drugs. This spreadsheet has been utilized in cases of pulmonary fibrosis associated with antiarrhythmics, cutaneous reactions associated with sulphonamides, anticonvulsants, foetal alcohol syndrome, and benzodiazepine withdrawal [48]. The spreadsheet requires five types of data, including pure data lines, input lines, assumption lines, estimation lines, and output lines. The MacBARDI tool is designed to update case analyses as new information becomes available, and it includes all the necessary standards for a good causality assessment technique, such as explicitness and flexibility. The tool supports learning and modelling, and it reduces the time required to evaluate cases [49].

Australian Method

The Australian Method is a probabilistic causality system that draws evidence from internal factors such as the timing of the reaction, nature of the disease, drugs, diet, and laboratory information from the case report [50]. It deliberately disregards any past knowledge of the suspected drug profile during the assessment process. This approach is the first of its kind and offers a unique perspective in determining the causal relationship between a drug and an adverse reaction. By focusing on internal factors, the Australian Method provides a thorough and objective evaluation of adverse drug reactions, which can inform effective pharmacovigilance practices [50].

Predictability assessment

Assessment of predictability is an important aspect of evaluating adverse drug reactions (ADRs). ADRs can be categorized into two types based on predictability: Type-1 (predictable) and Type-2 (unpredictable) ADRs [23,51]. Type-1 ADRs are more common and are related to the pharmacological effects of the drug. These reactions are often dose-dependent and can be anticipated as they are part of the known safety profile of the drug. For example, dryness of mouth caused by drugs with anticholinergic properties (like atropine, benztropine, etc) is a type-1 ADR. On the other hand, type-2 ADRs are rare and unpredictable, and they are not related to the pharmacological effects of the drug. These reactions are often caused by individual susceptibility or immune response to the drug. For instance, anaphylactic shocks caused by penicillin or chloramphenicol-induced aplastic anaemia are type-2 ADRs. Predictability assessment of ADRs is crucial in distinguishing between these two types of reactions and helps in understanding the safety profile of a drug [52,53].

Adverse event following immunisation

The World Health Organization (WHO) defines an adverse event following immunisation (AEFI) as any negative medical occurrence that happens after receiving a vaccine and may not have a direct causal relationship with the vaccine's use. The event may manifest as an unintended sign, abnormal laboratory finding, symptom, or disease [54]. 

Adverse events following immunisation (AEFI) can be categorized into five entities. The first is a vaccine product-related reaction, which is caused by one or more of the vaccine product's inherent properties. The second is a vaccine quality defect-related reaction, which is caused by one or more quality defects in the vaccine product, including the administration device. The third is an immunisation error-related reaction, which results from inappropriate vaccine handling, prescribing, or administration and is preventable. The fourth is an immunisation anxiety-related reaction or immunisation stress-related response (ISRR), which arises from anxiety about the immunisation. The fifth is a coincidental event, which is caused by something other than the vaccine product, immunisation error, or immunisation anxiety [54].

AEFI are usually reported through passive surveillance when compared to active surveillance systems. Vaccines/vaccination-related adverse events can reduce confidence in vaccines and may affect immunization coverage and disease incidence; hence, they must be dealt with rapidly and effectively. Timely reporting of AEFI followed by the appropriate and detailed investigation in important. The information and evidence that is collected during a good quality AEFI investigation hold the key for a successful evaluation of the event, and the circumstances of its occurrence and provide vital clues for the probable cause of its occurrence [54].

Severity assessment scale

Hartwig and Siegel's Severity Assessment scale is a tool that evaluates the severity of ADRs and has become widely used in clinical research and practice [55]. It was developed by Hartwig and Siegel in 1990 and consists of a five-point grading system ranging from mild to fatal. The grading system is based on the impact of the ADR on the patient's clinical status and the need for intervention. The scale's grading system ranges from mild, moderate, severe, and life-threatening, to fatal [55]. The severity of ADRs is determined by evaluating the need for treatment, changes in therapy, the impact on the patient's clinical status, and the urgency of medical attention needed. The severity grading helps healthcare providers prioritise interventions and inform clinical decision-making. ADRs are considered mild if they fall into levels 1 or 2, moderate if they are in levels 3 or 4, and severe if they are in levels 5 to 7. The seventh level is reserved for lethal reactions. This scale helps clinicians take prompt medical or interventional action to address any harm caused by the ADR [55]. 

Despite its usefulness, Hartwig and Siegel's Severity Assessment scale has limitations. It is subjective and relies on the clinician's interpretation of the patient's clinical status. Therefore, it may be influenced by the clinician's experience and expertise. Also, the scale does not consider the impact of ADRs on the patient's quality of life, which may be significant even in cases of mild ADRs. In conclusion, the Hartwig and Siegel's Severity Assessment scale is a valuable tool in assessing the severity of ADRs. However, it should be used alongside other tools to evaluate the impact of ADRs on patient health and quality of life. Its five-point grading system is based on the need for intervention and the impact on the patient's clinical status. The scale helps healthcare providers prioritise interventions and inform clinical decision-making. The levels used in this scale are shown in Table 9.

**Table 9:** Hartwig and Siegel’s severity assessment scale Mild: Level 1 or 2, Moderate: Level 3 or 4, Severe: Level 5, 6 or 7.

Hartwig and Siegel’s Severity Assessment Scale	
Level 1	An ADR occurred but required no change in treatment with the suspected drug
Level 2	The ADR required that treatment with the suspected drug be held, discontinued, or otherwise changed. No antidote or other treatment requirement was required. No increase in length of stay (LOS)
Level 3	The ADR required that treatment with the suspected drug be held, discontinued, or otherwise changed. AND/OR An Antidote or other treatment was required. No increase in length of stay (LOS)
Level 4	Any level 3 ADR which increases the length of stay by at least 1 day. OR The ADR was the reason for the admission
Level 5	Any level 4 ADR which requires intensive medical care
Level 6	The adverse reaction caused permanent harm to the patient
Level 7	The adverse reaction either directly or indirectly led to the death of the patient

Preventability assessment scale

The Schumock and Thornton Preventability Assessment Scale (S-T PAS) is a tool developed by Schumock and Thornton in 1992 to evaluate the preventability of adverse drug reactions (ADRs) [56]. This tool has become widely used in clinical research and practice. The S-T PAS consists of six categories that assess the preventability of ADRs. These categories include medication appropriateness, dosing and duration, drug-drug interactions, patient allergies and sensitivities, monitoring, and administration errors. Each category is assigned a score, and the total score determines the preventability of the ADR. The higher the score, the more preventable the ADR. The S-T PAS helps healthcare providers to identify areas for improvement in medication management, such as medication selection, dosing, and monitoring. It also helps to evaluate the impact of medication errors and implement strategies to prevent future errors. However, the S-T PAS has limitations. It requires a comprehensive understanding of the patient's medical history and medication regimen, which may not always be available. Additionally, it does not consider other factors that may contribute to the preventability of ADRs, such as patient adherence to medication regimens.

The modified form of the preventability assessment tool is commonly used nowadays [57,58]. It assesses whether the adverse drug reaction (ADR) is preventable and classifies it into three categories: definitely preventable, probably preventable, and non-preventable. The tool consists of two sections: section A with five questions and section B with four questions. The answers are either "yes" or "no". If any answer in section A is "yes", the ADR is classified as "definitely preventable". If all answers in section A are "no", then section B is used. If any answer in section B is "yes", the ADR is classified as "probably preventable". If all answers in section B are "no", then section C is used, and the ADR is classified as "non-preventable". The questions for all three sections are listed in Table 10.

**Table 10:** Preventability assessment (Schumock and Thornton criteria)

Preventability assessment (Schumock and Thornton criteria)
Section A (Answer in either yes or no)
Was there a history of allergy or a previous reaction to the drug? Was the drug involved was inappropriate for the patient's clinical condition? Was the dose, route, or frequency of administration was inappropriate for patient's age, weight or disease state? Was toxic serum drug concentration (or lab monitoring test) documented? Was there a known treatment for ADEs?
Section B (Answer in either yes or no)
Was required therapeutic drug monitoring or another necessary laboratory test not performed? Was the drug interaction involved in ADEs? Was poor compliance involved in ADE? Were preventative measures not prescribed or administered to the patient?
Section C
If all the above criteria are not fulfilled

## Conclusions

Reporting adverse drug reactions (ADRs) is imperative not only for ensuring optimal patient care but also for the generation of signals that contribute to drug safety monitoring and regulatory decision-making. In undertaking this review, our objective was to provide a comprehensive overview of the diverse methods available for the causality assessment of ADRs, aiming to elucidate their respective benefits and limitations. While widely utilized in clinical practice, the WHO causality assessment scales and the Naranjo probability scale are often favoured due to their accessibility and familiarity. However, despite their widespread use, challenges persist, including issues related to reproducibility, sustainability, and validity. It is evident that no single causality assessment tool has achieved universal acceptance across all clinical contexts, highlighting the ongoing need for research and innovation in this critical area of pharmacovigilance.
